# An updated review of case–control studies of lung cancer and indoor radon-Is indoor radon the risk factor for lung cancer?

**DOI:** 10.1186/s40557-016-0094-3

**Published:** 2016-03-03

**Authors:** Seungsoo Sheen, Keu Sung Lee, Wou Young Chung, Saeil Nam, Dae Ryong Kang

**Affiliations:** Department of Pulmonary and Critical Care Medicine, Ajou University School of Medicine, Suwon, Republic of Korea; Department of Humanities and Social Medicine, Ajou University School of Medicine, Suwon, Republic of Korea

**Keywords:** Lung cancer, Indoor, Radon, Case–control

## Abstract

Lung cancer is a leading cause of cancer-related death in the world. Smoking is definitely the most important risk factor for lung cancer. Radon (^222^Rn) is a natural gas produced from radium (^226^Ra) in the decay series of uranium (^238^U). Radon exposure is the second most common cause of lung cancer and the first risk factor for lung cancer in never-smokers.

Case–control studies have provided epidemiological evidence of the causative relationship between indoor radon exposure and lung cancer. Twenty-four case–control study papers were found by our search strategy from the PubMed database. Among them, seven studies showed that indoor radon has a statistically significant association with lung cancer. The studies performed in radon-prone areas showed a more positive association between radon and lung cancer. Reviewed papers had inconsistent results on the dose–response relationship between indoor radon and lung cancer risk.

Further refined case–control studies will be required to evaluate the relationship between radon and lung cancer. Sufficient study sample size, proper interview methods, valid and precise indoor radon measurement, wide range of indoor radon, and appropriate control of confounders such as smoking status should be considered in further case–control studies.

## Background

Lung cancer is a leading cause of cancer-related death in the world. Smoking is definitely the most important risk factor for lung cancer. Environmental and occupational exposure to carcinogens such as asbestos, radon, polycyclic aromatic hydrocarbons, and metals including nickel, arsenic, and chromium is also an important risk factor for lung cancer.

Radon (^222^Rn) is an inert gas produced naturally from radium (^226^Ra) in the decay series of uranium (^238^U). Radon gas is an important source of ionizing radiation, and it decays with a half-life of 3.8 days. Radon emanates from rocks, soils, and groundwater. It can damage the DNA of the respiratory epithelium, and exposure to radon is assumed to be the cause of lung cancer [1].

In 1988, the International Agency for Research on Cancer declared that radon is an important cause of lung cancer [2]. Radon exposure is strongly related to small cell carcinoma and squamous cell carcinoma of the lung [3–6]. Radon exposure is the second most common cause of lung cancer and the first risk factor for lung cancer in never-smokers [7].

Early studies on radon as a risk factor for lung cancer focused on occupational exposure such as in underground miners [8–12]. These studies established the increased risk of lung cancer for both smokers and non-smokers. After that, research on the relationship of indoor radon and lung cancer in the general population was conducted.

The main epidemiological evidence is from cohort studies or case–control studies. The cohort studies have a limited role in assessment for radon as a risk factor for lung cancer due to the relatively low incidence of lung cancer in the general population. To overcome the limitations of cohort studies, case–control studies play an important role in the assessment of the causative relationship between indoor radon exposure and lung cancer.

This review focuses on the case–control studies on the risk of lung cancer from indoor radon exposure in the general population. We intend to propose a desirable design of case–control studies on indoor radon and lung cancer risk for the general population in future studies.

### Literature search

**Fig. 1:**
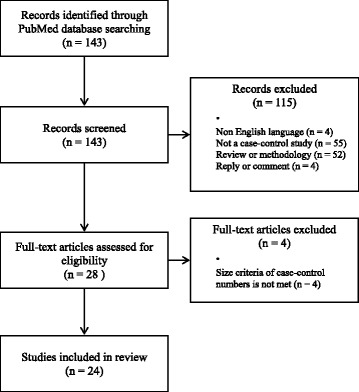
Flowchart for inclusion and exclusion

We performed searches for case–control studies about indoor radon exposure and lung cancer risk. The predefined keywords (indoor or residential; radon; case control; lung cancer) were used for search. The searches for the studies found 143 results through PubMed database. There were no language or time period limitations (up to 31st December 2015) during initial search. The identified studies were screened by two researchers independently. The studies other than case–control study excluded from this review. The language (English full-text) and size criteria (more than 100 case and 100 controls) restriction were adopted. Finally, 24 papers fulfilled the inclusion criteria. All of studies performed indoor air radon concentration measurements (Fig. 1).

### Case–control studies that included both smokers and never-smokers

Twenty studies that included both smokers and never-smokers were conducted (Table 1). There were inconsistencies in the study results from study to study on the relation between indoor radon and lung cancer risk. Sixteen studies showed an estimated odds ratio of 1 or more. Among them six studies showed statistically significant results.

**Table 1:** Case–control studies for radon and lung cancer in smokers and never-smokers

Author/year	Nation	Gender	Case (*n*)	Control (*n*)	Odds ratio^a^ (95 % CI)	Mean/median of indoor radon level (Bq/m^3^)		Number of alpha track detector used	Places of detector	Duration of radon measurements (months)	Interview type
						Case	Control				
Blot et al. 1990	China	Female	308	356	0.7 (0.4–1.3)	85		2	bedroom, living room	12	study subjects, proxy
Schoenberg et al. 1990	United States	Female	433	402	4.2 (0.99–17.5)	NA^b^		2	bedroom, basement	12	study subjects, proxy
Pershagen et al. 1992	Sweden	Female	210	209	1.7 (1–2.4)	128		2	bedroom, living room	3	study subjects, proxy
Létourneau et al. 1994	Canada	Both	738	738	0.77 (0.34–1.73)	120		2	bedroom, basement	12	study subjects, proxy
Pershagen et al. 1994	Sweden	Both	1360	2847	1.8 (1.1–2.9)	107		2	bedroom, living room	3	study subjects, proxy
Auvinen et al. 1996	Finland	Both	517	517	1.15 (0.69–1.93)	103	96	1	bedroom or living room	12	study subjects, proxy
Ruosteenoja et al. 1996	Finland	Male	291	495	1.5 (0.8–2.9)	213		1	living room or bedroom	12	study subjects, proxy
Darby et al. 1998	United Kingdom	Both	960	3126	1.79 (0.74–4.33)	58	56	2	bedroom, living room	6	study subjects
Alavanja et al. 1999	United States	Female	247	299	0.71 (0.3–1.3)	57	60	2	bedroom, kitchen	12	study subjects, proxy
Field et al. 2000	United States	Female	413	614	1.79 (0.99–3.26)	100	89	3	basement, first floor, second floor	12	study subjects
Pisa et al. 2001	Italy	Both	138	291	1.0 (0.3–3.1)	NA		1	bedroom	12	study subjects, proxy
Barros-Dios et al. 2002	Spain	Both	163	241	2.96 (1.29–6.79)	75	66	1	bedroom	3	study subjects, proxy
Wang et al. 2002	China	Both	768	1659	1.58 (1.1–2.3)	230	222	2	bedroom, living room	12	study subjects, proxy
Baysson et al. 2004	France	Both	486	984	1.11 (0.59–2.09)	83	80	2	bedroom, living room	6	study subjects
Bochicchio et al. 2005	Italy	Both	384	405	2.89 (0.45–18.6)	113	102	2	bedroom, living room	6	study subjects
Wichmann et al. 2005	Germany	Both	2963	4232	1.4 (1.03–1.89)	61	60	2	bedroom, living room	12	study subjects
Sandler et al. 2006	United States	Both	1474	1911	1.00 (0.93–1.07)	40	45	2	bedroom, lowest living level	12	study subjects
Thompson et al. 2008	United States	Both	200	397	2.5 (0.47–13.46)	68	66	2	bedroom, living room	12	study subjects, proxy
Wilcox et al. 2008	United States	Both	561	740	0.76 (0.36–1.61)	46	46	1	living room	12	study subjects, proxy
Barros-Dios et al. 2012	Spain	Both	349	513	2.21 (1.33–3.69)	NA		1	unclear	3–6	study subjects

^a^The highest exposure versus. the lowest exposure of radon

^b^Not applicable

The largest scale case–control study was a German study published in 2005. Wichmann et al. included study subjects aged 24 to 75 years comprising 2,963 cases and 4,232 controls [13]. The exposures to indoor radon were categorized into four groups with cut-off points at 50, 80, and 140 Bq/m^3^. The odds ratio (OR) for the category with indoor radon concentration between 80 and 140 Bq/m^3^ was 1.06 (95 % CI: 0.87–1.30), and the OR for the group with indoor radon concentration above 140 Bq/m^3^ was 1.40 (95 % CI: 1.03–1.89) compared to the category with the lower exposure (<50 Bq/m^3^). The level of 140 Bq/m^3^ is a meaningful radon concentration, as it is close to 148 Bq/m^3^, the EPA action level for radon in homes [7]. The risk for small cell carcinoma was higher than that for other histologies of lung cancer. This study showed a linear dose–response relationship between radon exposure and lung cancer in both smokers and never-smokers. The overall excess OR (EOR) of lung cancer per 100 Bq/m^3^ was 0.10 (95 % CI: −0.02–0.30). The EOR of lung cancer per 100 Bq/m^3^ was 0.14 (95 % CI: −0.06–0.52) in current smokers and 0.07 (95 % CI: −0.15–0.80) in never-smokers. This result suggests that the linear dose–response relation between radon exposure and lung cancer is more apparent in smokers than in never-smokers.

In 1990, Shoenberg et al. published a report on the strong association between indoor radon exposure and lung cancer risk [14]. This study was conducted in New Jersey, United States. They studied 433 female lung cancer cases and 402 controls. Both smokers and never-smokers were included in this study. Study subjects lived in the same house for at least 10 years to allow sufficient duration of indoor radon exposure. The OR of the category with the highest radon exposure (>148 Bq/m^3^) was 4.2 (95 % CI: 0.99–17.5) compared to the category with the lowest radon exposure (≤37 Bq/m^3^). Large cell undifferentiated carcinomas (*p* = 0.27) had a pattern of increasing risk with increasing radon concentration. This study reported stronger evidence of the lung cancer risk caused by indoor radon compared to other case–control studies. However, separate analyses for never-smokers failed to show the relation between radon exposure and lung cancer risk. There were 61 lung cancer cases of never-smokers in this study. Only two cases and six controls were never-smoking subjects exposed to the highest radon concentration. The possibility of inaccuracies in the exposure estimates, selection biases, and the small number of never-smoking subjects with high radon exposures should be noted in this data interpretation.

Some studies showed weak or non-significant evidence of the association between indoor radon exposure and lung cancer. In 2008, Wilcox et al. reported the results of a case–control study on radon and lung cancer in New Jersey, United States [4]. A total of 651 lung cancer cases and 740 controls contributed to the analysis of radon exposure and the risk of lung cancer. There were only 40 (6.1 %) never-smokers in the lung cancer cases and 116 (16 %) never-smokers in the controls. Indoor radon exposures were divided into six categories (≤25, 25–49, 50–74, 75–99, 100–149, and ≥150 Bq/m^3^). The authors failed to show a statistically significant relation between indoor radon exposure and lung cancer risk. However, the ORs showed a tendency to increase with elevated radon exposure up to the 100–149 Bq/m^3^ category. Although the EOR per 100 Bq/m^3^ was −0.13 (95 % CI: −0.14–0.56) for males and 0.29 (95 % CI: −0.12–1.70) for females, this study showed radon exposure slightly increased the risk of lung cancer (overall EOR = 0.05 per 100 Bq/m^3^, 95 % CI: −0.14–0.56).

The highest exposure category (≥150 Bq/m^3^) had few cases, and the ORs were smaller (OR 0.76, 95 % CI: 0.36–1.61) than those in the lower exposure categories. The results of this study seem to suggest a nonsignificant small effect of residential radon exposure on the risk of lung cancer. However, this study may underestimate the true exposure–response relationship due to selection bias among heavy smokers, influence for information by proxy interviews, and uncertainties regarding radon measurements.

In Asia, two case–control studies met our searching conditions. Both studies were conducted in China. These Chinese studies also showed inconsistent results on indoor radon and lung cancer risk.

In 1990, Blot et al. reported that there was no difference in indoor radon levels between lung cancer cases and controls [15]. This study included 308 lung cancer cases and 356 controls, and all study subjects were women. The OR of the category with the highest radon exposure (>296 Bq/m^3^) was 0.7 (95 % CI: 0.4–1.3) compared to the category with the lowest radon exposure (<74 Bq/m^3^). They also reported that lung cancer risk did not increase with increasing radon levels in homes in Shenyang City.

Contrary to the results of Blot et al., Wang et al. (2002) published a case–control study conducted in Gansu Province, China [16]. Gansu Province is a rural area with high radon levels and low mobility of residents. The OR of the category with the highest radon exposure (>300 Bq/m^3^) was 1.58 (95 % CI: 1.1–2.3) compared to the category with the lowest radon exposure (<100 Bq/m^3^). The EORs for indoor radon concentration of 100 Bq/m^3^ were 0.14 (95 % CI: −0.03–0.54), and this result showed that lung cancer risk increased with increasing indoor radon concentrations. The overall results of this study showed that high levels of indoor radon exposure increased the lung cancer risk. However, never-smokers were only 5 % of cancer cases and 8.9 % of controls in the male subjects in this study. The type of residence was an underground dwelling in more than half of both lung cancer cases and controls, and this pattern of residence is different from other studies.

### Case–control studies that included non-smokers only

Case–control studies that include non-smokers only are very important for the estimation of the indoor radon and lung cancer association, because the effect of smoking, the largest confounder, can be minimized. There were only four studies on indoor radon exposure and lung cancer in non-smokers (Table 2) [17–20]. Two studies included only female non-smokers, and the other two studies included both male and female non-smokers.

**Table 2:** Case–control studies for radon and lung cancer in non-smokers

Author/year	Nation	Gender	Case (*n*)	Control (*n*)	Odds ratio^a^ (95 % CI)	Median/mean of indoor radon level (Bq/m^3^)		Number of alpha track detector used	Places of detector	Duration of radon measurements (months)	Interview type
						Case	Control				
Alavanja et al. 1994	United States	Female	538	1183	1.2 (0.9–1.7)	67	67	2	bedroom, kitchen	12	study subjects, proxy
Lagarde et al. 2001	Sweden	Both	258	487	1.55 (0.88–2.73)	87	80	2	bedroom, living room	3	study subjects, proxy
Kreuzer et al. 2002	German	Female	234	535	NA^b^	45	44	2	bedroom, living room	12	study subjects
Torres-Durán et al. 2014	Spain	Both	192	329	2.42 (1.45-4.06)	NA		1	bedroom	3	study subjects

^a^The highest exposure versus. the lowest exposure of radon

^b^Not applicable

Lagarde et al. (2001) published the results of their study, which included 258 never-smoking lung cancer cases and 487 never-smoking controls [18]. Both males and females were included in the study. The mean periods of residence in radon-measured homes were 26.1 years among cases and 26.8 years among controls. The OR of the category with the highest radon exposure (>140 Bq/m^3^) was 1.55 (95 % CI: 0.88–2.73) compared to the category with the lowest radon exposure (≤50 Bq/m^3^). This study showed a dose–response effect of radon on lung cancer (overall ERR per 100 Bq/m^3^ = 0.28 (CI: −0.05-1.05)). The ERRs for the indoor radon concentration of 100 Bq/m^3^ were 0.02 (95 % CI: −0.06–0.32) in subjects not exposed to environmental tobacco smoke at home and 0.29 (95 % CI: −0.03–1.24) in subjects exposed to environmental tobacco smoke at home. These results also might suggest an effect of indoor radon exposure and environmental tobacco smoke on the risk of lung cancer.

Torres-Durán et al. (2014) studied 192 lung cancer cases and 329 controls in Galicia, Spain [17]. Study subjects were male or female never-smokers aged over 30 years. Adenocarcinoma was the most common histologic type of lung cancer (77.5 %) in this study. The median number of years living in the radon-measured home was over 30 years for both cases and controls. Indoor radon exposures were divided into four categories according to the level of radon measured (≤100, 101–147, 148–199, and ≥200 Bq/m^3^). This study showed a significant relation between indoor radon and lung cancer. The OR of the highest radon-exposed individuals (>200 Bq/m^3^) was 2.42 (95 % CI: 1.45–4.06) compared to the lowest radon-exposed group (<100 Bq/m^3^). In addition, individuals who had been exposed to >200 Bq/m^3^ and who had not lived with a smoker showed a significant OR of 1.99 (95 % CI: 1.16–3.41). Moreover, these results suggest an effect of indoor radon exposure and environmental tobacco smoke on the risk of lung cancer. However, this study could not evaluate the relation between radon and lung cancer subtype, because there were only 43 (22.5 %) subjects with histologic types of lung cancer other than adenocarcinoma.

However, a German case–control study by Kreuzer et al. (2002) failed to show a link between indoor radon and lung cancer risk in non-smoking women [19]. They defined non-smoking women as having smoked less than 200 cigarettes in a lifetime. This study included 234 lung cancer cases and 535 controls. Measurements of one-year radon concentrations in the last dwelling were performed. The median concentrations of indoor radon did not differ between lung cancer cases (45 Bq/m^3^) and controls (44 Bq/m^3^). There was no significant trend in lung cancer risk with increasing indoor radon levels (*p* = 0.22). However, 42 % of cancer cases and 16 % of controls were excluded from analysis due to missing radon measurements in this study.

Alavanja et al. (1994) published their study that included never-smokers and former smokers [20]. This study was carried out in Missouri, United States. Former smokers were defined as women who ceased using tobacco 15 or more years prior to the interview. Radon exposures were divided into five categories by quintile interval (0.1–0.79, 0.80–1.19, 1.20–1.69, 1.70–2.45, 2.46–15.3 pCi/L). The relative risk of lung cancer for women exposed to the highest radon concentrations was 1.20 (95 % CI: 0.7–1.7) compared with the women exposed to the lowest radon concentrations. A positive dose–response relation was suggested for adenocarcinoma among directly interviewed women (*p* = 0.04 categorical data analysis). However, the relative risk for the highest decile of radon exposure was below 1.0 compared with the lowest decile. An association between indoor radon and lung cancer risk was not convincingly demonstrated in this study. However, proxy interviews were conducted in 341 cases (63 %). There was a limited range of radon concentrations, because Missouri is an area with relatively lower radon exposure. The cut-off point of the highest radon exposure category was only 91 Bq/m^3^, and this was the lowest cut-off point of the highest radon exposure category except for the study by Sandler et al. (2006) (53 Bq/m^3^) [21]. These limitations could have influenced the results of this study.

### Studies in radon-prone areas versus lower exposure areas

The studies in radon-prone areas had a tendency to show an association between radon and lung cancer risk. However, the studies that failed to show a relation between indoor radon exposure and lung cancer risk were usually conducted in areas with relatively lower radon exposure.

Blot et al. (1990) conducted a case–control study in the northern industrial city of Shenyang, and this city is an area with lower radon exposure in China [15]. Their results failed to show a relation between indoor radon and lung cancer. Another Chinese study by Wang et al. (2002) was carried out in Gansu Province, a rural area with high radon levels [16]. This study provided evidence that high levels of residential radon increase the risk of lung cancer. According to the results from Wang et al., the mean indoor radon concentrations for cases and controls were 230.4 and 222.2 Bq/m^3^, respectively, and these radon levels were much higher than the results from Blot et al. (85 Bq/m^3^).

A German study by Kreuzer et al. (2002) and American studies by Alavanja et al. (1994) and by Sandler et al. (2006) were conducted in areas with lower radon exposure, and these studies also failed to show an association between indoor radon exposure and lung cancer risk [19, 21].

Sandler et al. divided indoor radon exposures into four categories by quartiles of radon exposure. The cut-off points for the highest and lowest quartiles of indoor radon concentrations were only 53 and 18 Bq/m^3^, respectively, and these cut-off points were lower than those of all other studies. This study was conducted in Connecticut and Utah, areas with lower radon exposure in the United States, and this could be one of the main reasons it failed to show a relation between indoor radon and lung cancer.

Contrary to the results by Sandler et al., a case–control study (Field et al., 2000) conducted in Iowa, a radon-prone area in the United States, showed significant results for radon and lung cancer risk [22].

Recent case–control studies have shown strong effects of indoor radon exposure on lung cancer except for two studies (Sandler et al. 2006, Wilcox et al. 2008) in the United States [4, 21].

Spanish studies in 2012 and 2014 were carried out in a radon-prone area (Galicia) [17, 23]. About 20 % of all dwellings were above the EPA action level for indoor radon, because the ground in Galicia contains high levels of granite [7]. These studies showed significant evidence of the link between indoor radon and lung cancer risk.

Radon exposure occurs both indoors and outdoors. Indoor radon exposure is higher in radon-prone areas than in lower radon exposure areas. Additional radon exposure from the environment rather than indoor radon is much higher in radon-prone areas than in lower radon exposure areas. Thus, even if we consider different levels of indoor radon between radon-prone areas and lower radon exposure areas, dwellers in radon-prone areas are actually exposed to much larger amounts of radon than those in lower radon exposure areas due to the accumulation of indoor and outdoor radon exposure. The threshold dose of radon for a carcinogenic effect could be more easily accumulated and shorter periods could be required for radon to become a carcinogenic hazard in radon-prone areas. In addition, studies in radon-prone areas might have an advantage for the evaluation of the dose–response relationship of indoor radon and lung cancer risk, because the radon exposure range is wider than in lower radon exposure areas. Therefore, this geological characteristic could have advantages in analyzing the radon effect on lung cancer risk.

### A dose–response relationship between indoor radon and lung cancer

Nine reports showed evidence that lung cancer risk increases with increasing indoor radon concentration (Table 3).

**Table 3:** Dose–response effects of radon for lung cancer

Author/year	Nation	EOR^a^/ERR^b^ per 100 Bq/m^3^ (95 % CI) radon concentration			
		Overall	Smokers	Ex-smokers	Non-smokers
Pershagen et al. 1994	Sweden	0.10 (0.01–0.22)	0.14 (−0.06–0.52)	NA	0.07 (−0.15–0.80)
Darby et al. 1998	United Kingdom	0.12 (−0.05–0.33)	−0.04 (−0.22–0.14)	0.19 (0.03–0.35)	0.04 (−0.49–0.57)
Field et al. 2000	United States	0.24 (−0.05–0.92)	NA^c^	NA	NA
Lagarde et al.^d^ 2001	Sweden	0.28 (−0.05–1.05)			0.02 (−0.06–0.32) not exposed to ETS^e^ at home
					0.29 (−0.03–1.24) exposed to ETS at home
Wang et al. 2002	China	0.19 (−0.05–0.47)	I:0.34/II:0.02/III:0.80^f^		0.09
Baysson et al. 2004	France	0.04 (−0.01–0.11)	NA	NA	NA
Bochicchio et al. 2005	Italy	0.14 (−0.11–0.46)	0.16 (−0.12–0.51)^g^		-0.23 (−0.64–0.66)
Wichmann et al. 2005	Germany	0.10 (−0.02–0.30)	0.14 (−0.06–0.52)	0.07 (−0.03–0.42)	0.07 (−0.15–0.80)
Wilcox et al. 2007	United States	0.05 (−0.14–0.44)	Male: −0.13 (CI: −0.14–0.56)		
			Female: 0.29 (CI: −0.12–1.70)		

^a^Excess odds ratio

^b^Excess relative risk

^c^Not applicable

^d^Studies that included only never-smokers

^e^Environmental tobacco smoking

^f^Smoking risk levels: I, other-light smokers; II, duration ≥30 years and amount ≥10 cigarettes/day; III, duration ≥ 40 years and amount ≥ 20 cigarettes/day

^g^Eversmoker

The lung cancer risk increases from 4 (Baysson et al. 2004) to 28 % (Lagarde et al. 2001) with increasing radon levels per 100 Bq/m^3^ in overall subjects. The lung cancer risk increases from 2 to 80 % with increasing radon levels per 100 Bq/m^3^ in smokers.

Darby et al. (1998) is the only study that did not show a positive dose–response relationship between radon and lung cancer in a subgroup analysis for smokers, although there were positive dose–response relationships between radon and lung cancer in both the non-smoker and ex-smoker subgroups. The authors stated that this study was carried out in Devon and Cornwall, South-west England, which is a radon-prone area in the United Kingdom. However, measured mean concentrations of indoor radon (cases 58 Bq/m^3^, controls 56 Bq/m^3^) were lower than those in the previous database in this area. Moreover, these concentrations were not significantly higher levels of indoor radon compared with other studies. The possibility that the study was actually conducted in a lower radon exposure area or that the radon measurements were underestimated should be considered.

The lung cancer risk increased from 4 to 28 % with increasing radon levels per 100 Bq/m^3^ in the subgroup analysis for never-smokers. Bochicchio et al. (2005) failed to show a positive dose–response relationship between indoor radon and lung cancer risk in never-smokers.

The results of the linear dose–response relationship between radon and lung cancer from smokers were much higher than the results from ex-smokers and non-smokers, and these suggest that indoor radon exposure has the strongest effects on lung cancer in smokers.

Among the studies that included only never-smokers as study subjects, only one study by Lagarde et al. (2001) showed a linear relationship between radon exposure and lung cancer risk [18]. The overall ERR of this study was the highest (ERR = 0.28, 95 % CI: −0.05–1.05) compared to the results from other studies. The ERR was much higher in subjects with environmental tobacco smoking exposure than in subjects without environmental tobacco smoking exposure. This result shows that exposure to environmental tobacco smoking at home also has strong effects on the effect of indoor radon exposure on lung cancer risk.

Previous studies on radon and lung cancer in miners showed a linear dose–response relationship for lung cancer risk and mortality [24, 25]. A European collaborative analysis study of the general population included 7,148 lung cancer cases and 14,208 controls and was published by Darby et al. in 2006 [26]. This study reported that the linear dose–response relationship remained significant with no evidence of a threshold when only individuals with radon concentrations of < 200 Bq/m^3^ were included. A recent meta-analysis on this issue reported evidence of a nonlinear dose–response relationship between radon exposure and the risk of lung cancer [27]. Duan et al. (2015) concluded that there is a non-linear dose–response relationship between indoor radon and lung cancer risk, but according to the results, a linear dose–response relationship was obvious in the individuals exposed to less than 200 Bq/m^3^ of radon, but this incremental relationship was weak in individuals exposed to more than 200 Bq/m^3^ of radon. However, the heterogeneity of these studies causes some limitations in data interpretation.

There were no consistent results on the dose–response relationship between indoor radon and lung cancer risk. As the dose of exposure for indoor radon is relatively smaller than that for miners, it is difficult to evaluate the dose–response effect of indoor radon on lung cancer risk. Further studies are needed to evaluate the dose–response relationship between indoor radon and lung cancer risk.

### Radon and lung cancer risk of histologic subtype

Shoenberg et al. (1990) showed a strong association between indoor radon exposure and lung cancer risk [14]. This study reported that large cell undifferentiated carcinomas (*p* = 0.27) have a pattern of increasing risk with increasing radon concentrations. The study by Alavanja et al. (1994) that included never-smokers and former smokers showed a positive dose–response relation for adenocarcinoma among directly interviewed women (*p* = 0.04 categorical data analysis) [20]. The risk for small cell carcinoma was significantly elevated (EOR = 0.29 CI: 0.04–0.78) in the study by Wichmann et al. (2005). Wilcox et al. (2008) failed to show a statistically significant relation between indoor radon exposure and overall lung cancer risk [4]. However, the authors showed that radon exposure had a strong effect on small cell lung cancer in cases of both genders, but the causative relation of indoor radon and squamous cell carcinoma was only demonstrated in male cases.

Previous studies have showed evidence that indoor radon exposure is strongly related to small cell carcinoma and squamous cell carcinoma of the lung [28, 29]. The studies that only included never-smokers were very limited and produced inconsistent data for radon and adenocarcinoma of the lung [6, 30]. Further evaluation of the causative relation of indoor radon and subtypes of lung cancer, especially adenocarcinoma, is required.

### Future study design

Smoking is the leading cause of lung cancer. To maximally control the strong effects of smoking on lung cancer, a substantial number of never-smokers should be included in future studies. Chronic radon exposure is required to cause lung cancer in the general population. The induction duration of lung cancer due to radon exposure is usually from 5 to 25 years [31]. However, measuring indoor radon for this long is practically impossible, so we need to estimate indoor radon concentrations exactly over these periods using radon measurement with time-weighted averages for relatively short durations. However, the indoor radon concentrations of the same house can vary with the house’s ventilation pattern, the season, and the year [32]. It is more reasonable to measure indoor radon concentrations for a yearlong period than for a season or a shorter period of a year. If possible, repetition of a yearlong measurement of indoor radon is recommended. Urban residents are more mobile than rural residents. Thus, a single measurement of indoor radon for the last dwelling may be inappropriate. Indoor radon measurements must not be localized in the house, but further measurements must be considered in the workplace and on public transportation such as subways.

## Conclusions

Although some reports failed to show indoor radon as a lung cancer risk, radon is an obvious cause of lung cancer if we consider previous studies and scientific evidence. The relatively low radon exposures in houses and low incidence of lung cancer in the general population, as well as the strong carcinogenic effects of smoking and other environmental materials, make estimating the real risk of indoor radon on lung cancer difficult in epidemiological studies.

To overcome the limitations of epidemiological studies, case–control studies will be essential. To conduct a case–control study successfully, appropriate study design is very important. Large samples will be required to detect differences between cases and controls and enable precise estimations of the effect of radon on lung cancer risk. Controls should be as similar as possible in terms of age, sex, social/economic status, area of residence, and housing type to their matched lung cancer cases. To minimize the effect of smoking, studies in never-smokers are very important. Case–control studies on never-smokers could be conducted to evaluate the synergistic effect of environmental tobacco smoking and indoor radon on lung cancer risk. However, studies including both smokers and never-smokers are also required to evaluate the interactions between radon and smoking for smoking-related lung cancer. Comprehensive face-to-face interviews are needed to exclude the influence of inaccurate data by proxy interviews. Exact estimations of indoor radon exposure are also required. To get wider ranges of radon exposure and to evaluate the dose–response relation between indoor radon and lung cancer risk more accurately, the study area should be a radon-prone area rather than an area with lower radon exposure. Further studies also need to evaluate the different effects of radon on histologic subtypes of lung cancer.
