# Erratum to: An updated review of case–control studies of lung cancer and indoor radon-Is indoor radon the risk factor for lung cancer?

**DOI:** 10.1186/s40557-016-0155-7

**Published:** 2016-12-28

**Authors:** Seungsoo Sheen, Keu Sung Lee, Wou Young Chung, Saeil Nam, Dae Ryong Kang

**Affiliations:** 1 Department of Pulmonary and Critical Care Medicine, Ajou University School of Medicine, Suwon, Republic of Korea; 2 Department of Humanities and Social Medicine, Ajou University School of Medicine, Suwon, Republic of Korea

## Erratum

In this version of this article that was originally published [1] the Acknowledgements section was missing and is included correctly below:

Acknowledgements

This subject is supported by Korea Ministry of Environment (MOE) as “the Environmental Health Action Program” (Grant Number 2015001350002).

The publisher apologises for these errors.
